# The “100,000+ Clicks” Dream Comes True: A Study on the Click-and-Read Behavior of WeChat Tweets From the Perspective of Emotional Expression

**DOI:** 10.3389/fpsyg.2021.739240

**Published:** 2021-10-28

**Authors:** Jun Fan, Tingting Chen, Li Lin

**Affiliations:** School of Business Administration, Zhejiang Gongshang University, Hangzhou, China

**Keywords:** WeChat public accounts, tweet titles, emotional expressions, click-and-read behavior, the limited attention capacity model

## Abstract

An in-depth and refined empirical study on the emotional expression of information and the information processing mechanism of audiences is carried out to provide enterprises and other organizations with insights and references as regard to the effective utilization of WeChat Tweets for information dissemination and marketing purposes. Based on 1,465 actual tweets from two different types of WeChat public accounts (knowledge communication and information releasing), this paper applies the limited attention capacity model and the signaling theory to analyze the influence of emotional presence, emotional complexity, emotional intensity, and emotional polarity of tweet titles on the click-and-read behavior of the audience. The results show that for WeChat public accounts serving the purpose of knowledge communication, emotional presence and emotional complexity of tweet titles, as well as the emotional intensity of positive tweet titles, has no significant effect on the click-and-read behaviors of the audience. Besides, the emotional intensity of negative tweet titles has a significant negative impact on the audience’s click-and-read behaviors. While for WeChat public accounts serving the purpose of information releasing, tweet titles with emotional presence and lower level of emotional complexity are more likely to trigger click-and-read behaviors of audiences; emotional intensity of negative tweet titles has no significant effect on the click-and-read behaviors of audiences, and emotional intensity of positive tweet titles has a significant negative impact on the audience’s click-and-read behaviors. Thus, this study further analyzes the influence of emotional factors, such as emotional existence, emotional complexity, emotional intensity, and emotional polarity of tweet titles on the click-and-read behavior of consumers and further explores the emotional information processing mechanism of WeChat tweet readers.

## Introduction

WeChat is a leading “super app” in China, with a variety of technical functions, including video and voice calling, payment, online mall, loan, and entertainment. In 2012, WeChat added the service of “WeChat public account” on the basis of its original functions, allowing users to open accounts and share original articles (Su and Xiao, 2020). As of the end of 2020, the number of public account platforms has exceeded 20 million in China, and the monthly active population on WeChat has exceeded 1.2 billion, which accounts for 86% of the total population (Statista.com, 2020).

The prevalence of WeChat public account has changed the way in which people in China acknowledge, perceive, and influence the world. The rapidly emerging mobile Internet has had a profound impact on the way information is communicated and on people’s cognitive activities and social behaviors. On one hand, people are offered unprecedentedly convenient access to reading and socializing regardless of their levels of education, income, or occupation. A mobile device at hand equips anyone, anytime, and anywhere, with the ability to quickly scan or forward real-time materials through social media, such as WeChat. On the other hand, a large number of enterprises, organizations, and individuals are publishing massive amounts of information to the audience in real time through online media, such as the WeChat public account, which has caused a phenomenal amount of “information overload.”

The massive volume of online information, diversified choices of media, and fast-paced lifestyles have significantly changed people’s reading habits, signaling the arrival of a “title-reading era.” People have become so occupied with the overwhelming explosion of information that they are having difficulty finding the time to spend reading details of every article. Subsequently, titles have become the most essential, or even major, source of information for the audience before they have time to further screen and acquire information; it is just like the way when people pick a book only because they are attracted to its cover or choose a newspaper by scanning the headlines. To catch the eyes of the readers, various WeChat public accounts have also adopted short text and emotional expressions in tweet titles to stimulate the audience’s click-and-read behaviors and interest in reading. For a tweet on a WeChat public account with a reading volume of “100,000+,” the emotional expression of its title plays an undeniable role in attracting viewers’ attention.

With the popularity and prevalence of social media platforms such as WeChat and Weibo, issues, such as acquiring, analyzing, and disseminating information through these platforms, have begun to attract academic attention. Existing research on issues, such as network information dissemination, mainly focuses on the approaches (Zhang, 2017; Fadel et al., 2021), spreading channel features (Baccarella et al., 2018; Talwar et al., 2019), and dissemination process (Jang et al., 2018; Domenico et al., 2021) and so on. Text analysis research on network information is conducted, which mainly involves network public opinion analysis (Li et al., 2020; Zhao et al., 2020), analysis of information expression method (Wang et al., 2012), and text sentiment analysis (Zhen et al., 2014; Tago and Jin, 2018), as well as methods of text analysis (Anjaria and Guddeti, 2014; Cao et al., 2018) and so on. The research on network information reading behavior from the perspective of emotion mainly focuses on network information sharing, such as the action path of network information sharing (Kissler et al., 2007; Li and Liu, 2018), the difference in the effect of various emotions on network information sharing (Tracy and Randles, 2011; Liu et al., 2015), and the influence of emotional valence on network information sharing (Berger and Milkman, 2012; Zhu et al., 2014). Research on the reading behaviors of socialized media information from the emotional perspective is still rare, with existing research mostly focusing on the dissemination of emotional information. Zhao et al. (2012) divide emotions into four basic categories (anger, joy, sadness, and disgust) in their research on the spread of Chinese tweets in Weibo (a Twitter-like online social network in China) and find that anger is more easily spread in social media. Feng and Tang (2017) take WeChat public account tweet as an example and analyze the relationship between the emotional expression of the tweet content and its communication effect.

This paper studies the WeChat public account, the most popular social media in China, and explores tweet titles in the WeChat public accounts based on the perspective of emotional expression. By referring to the limited attention capacity model and the signaling theory, we utilize real data from the backstage of two different types of public accounts (knowledge communication and information releasing) and carry out specific theoretical and empirical analysis as regard to the influence of emotional information on the click-and-read behavior of the audience of the two different types of public accounts based on such dimensions as emotional presence, emotional complexity, emotional intensity, and emotional polarity of tweet titles. This article further enriches and expands the research field of social media marketing through deepening and refining the research on issues, such as the emotional expression of information and the mechanism of information processing of tweet readers. Our study provides insights and practical guidance on the effective use of WeChat public account tweets for the purpose of information dissemination and marketing.

## Theoretical Basis and Research Hypotheses

### The Limited Attention Capacity Model and the Signaling Theory

The basic assumption of the limited-capacity model of attention is that an individual’s ability to process information is limited, and tasks with higher cognitive requirements require more attention resources (Xu and Chen, 2017; Zeng et al., 2019). As a result, when faced with different information, individuals will consciously or subconsciously allocate priorities as regard to information processing correspondingly, so as to selectively invest their cognitive resources. Also, there is a relatively significant difference in the intensity of attention (Wu et al., 2015). Lynch and Srull (1982) classify cognitive tasks into categories of primary tasks and secondary tasks according to their relative importance and point out that the attention capacity available for primary tasks is significantly greater than that for secondary tasks. Xu and Chen (2017) believe that the limited attention of individuals will, to a certain extent, limit the production of language-based information. Therefore, some trade-offs must be considered among factors such as the accuracy, fluency, and complexity of language used.

The signaling theory states that in the case of asymmetric information, the party lacking information will try to speculate on the authenticity of the information based on various signals exhibited by the information provider (Moorthy, 2012; Huang et al., 2020). For example, users who are exposed to a product for the first time often know very little about the product, so they will guess the true quality of the product through relevant information that is available to them (Hou et al., 2014). Some researchers point out that under the situation of asymmetric information between buyers and sellers, buyers will actively search for signals that can help them distinguish the quality of products or services (Huang et al., 2016; Johnson et al., 2021). On the other hand, sellers will actively pass on signals that can produce positive effects and even intentionally provide false signals. Xiao et al. (2014) and Peng and Mi (2017) believe that there are three primary actors involved in the signal theory: the signaler, the signal itself, and the receiver. A signaler is generally an insider who has all the information, and the receiver is usually an outsider who lacks information but needs that information.

### The Influence of the Emotional Presence of Tweet Titles on the Click-and-Read Behaviors of the Audience in Different Types of WeChat Public Accounts

Schoefer and Ennew (2005) point out that emotions are thought to have an important role in processes related to the audience’s capacity in attention-paying, memory construction, and decision-making. Phelps et al. (2006) find that because the information presented with emotion can produce a certain degree of “emotional stimulus,” people’s sensitivity in discerning emotional information is significantly higher than that in discerning information without emotional content. Gray et al. (2013) and Schmidt et al. (2014) also believe that individuals show improvement in information processing when faced with stimulation from emotional information as compared with the case when they are faced with non-emotional information, and thus, they will invest more attention resources. Li and Liu (2018) point out that information containing emotion is more communicative than information without emotion. According to both the limited attention capacity model and the signaling theory, in the face of a large number of tweets from the WeChat public account, audiences with limited information processing capability usually put their attention first on the titles of the tweets. However, the amount of information conveyed in the titles is very limited and it is difficult for the audience to accurately judge whether the content of the tweet is worth reading. Therefore, audiences are put in a disadvantaged position as defined by information asymmetry. At this time, as a stimulus signal, the emotional information in the titles may become the focus of the audience and can affect their click-and-read behaviors.

Besides, there are many ways to classify WeChat public accounts, such as the ones based on the content (Huang and Wang, 2015), function (Wu et al., 2017), and brand (Zhu and Yuan, 2019) of the WeChat public accounts. Based on the differences of tweet content and target audience, existing WeChat public accounts can be divided into different types, including types such as the type of information releasing and the type of knowledge communication (Huang and Wang, 2015; Ou et al., 2015). Audiences who have an interest in different types of WeChat public accounts also show major differences in their motivation in reading and their focus on specific information (Ou et al., 2015). On one hand, the content of tweets from WeChat public accounts with the purpose of knowledge communication mostly involves the promotion and popularization of certain types of professional knowledge (e.g., Xiaomuchong. A WeChat official account that mainly publishes scientific research). Most of the audiences going through this type of public accounts have a high level of education and present more rational and pragmatic reading motives. Thus, they care more about the authenticity, professionalism, and practicality of the tweets. Therefore, emotional information in tweet titles is not often their major concern, and the presence or absence of emotion may not be a stimulating signal that triggers their click-and-read behaviors. On the other hand, the content of tweets from WeChat public accounts with the purpose of information releasing mainly involves the releasing or announcement of various news and information (e.g., Hangzhoufabu. A WeChat public account that mainly publishes news). The audience of this type of public account usually covers a wide range of population whose motivation of reading is mostly to satisfy curiosity or gain pleasure. For this type of audience, both the authenticity and the emotional and entertaining elements of the tweets are important. Therefore, the emotional information lying within the titles of these tweets often becomes their focus and the presence or absence of emotion is likely to be a stimulating signal for them to trigger click-and-read behaviors. Hence, the following assumptions are proposed:

*Hypothesis 1a: For WeChat public accounts serving the purpose of knowledge communication, the emotional presence of the tweet title has no significant effect on the click-and-read behaviors of the audience*.

*Hypothesis 1b: For WeChat public accounts serving the purpose of information releasing, tweet titles with the presence of emotion are more likely to trigger the click-and-read behaviors of the audience than those without the presence of emotion*.

### The Influence of Emotional Complexity of Tweet Titles on the Click-and-Read Behaviors of the Audience for Different Types of WeChat Public Accounts

Ekman (1993) divides human emotions into six categories: surprise, disgust, sadness, anger, fear, and joy. Xu et al. (2008) divide emotional vocabulary in Chinese into seven categories: joy, contentment, anger, sorrow, fear, disgust, and shock. The emotional complexity of the tweet title refers to the number of different emotional categories involved in the emotional vocabulary applied in the title; the larger the number, the higher the emotional complexity. Research of Miller (1976) found that less information exposure will improve the audience’s evaluation of stimuli, while excessive exposure will instead inhibit the formation of positive attitudes. The arousal theory points out that an individual’s perception of novel stimuli will gradually attenuate with the repeated presence of the stimuli, and excessive repetition of stimuli will reduce the level of novelty perceived. As a stimulating signal, the emotional vocabulary in the tweet title is also likely to demonstrate a similar attenuation effect. Also, according to the signaling theory, when the level of the emotional complexity of the tweet title is high (i.e., when there are many different types of emotional vocabulary present in the same tweet title), a variety of different stimulating signals will be sent to the audience at the same time, making it difficult for the audience to form judgments and make decisions. For WeChat public accounts with the purpose of knowledge communication, emotional information is not the main concern of the audience and the emotional complexity of the tweet title may not have an important impact on their click-and-read behaviors. For WeChat public accounts with the purpose of information releasing, multiple types of emotion-stimulating signals sent by tweet titles with high emotional complexity are likely to cause a reduction in the audience’s perception resulting in difficulty in making judgments and decisions or even causing doubts as regard to the authenticity of related tweets. Hence, the following assumptions are proposed:

*Hypothesis 2a: For WeChat public accounts serving the purpose of knowledge communication, the emotional complexity of the tweet title has no significant effect on the click-and-read behaviors of the audience*.

*Hypothesis 2b: For WeChat public accounts serving the purpose of information releasing, tweet titles with a lower level of emotional complexity are more likely to trigger the click-and-read behaviors of the audience than those with a higher level of emotional complexity*.

### The Interactive Influence of Emotional Intensity and Emotional Polarity of Tweet Titles on the Click-and-Read Behaviors of the Audience in Different Types of WeChat Public Accounts

Emotional intensity refers to the level of emotional response any given content or information could potentially generate; a strong emotional tendency will send signals to the audience and, in turn, get the audience’s attention (Zhen et al., 2014; Xing et al., 2018). Emotional polarity refers to the positive or negative tendency of emotional information (Skowronski and Carlston, 1989). In this study, emotional intensity and emotional polarity of the tweet title refer to the emotional tendency and intensity contained in the emotional vocabulary in the title. According to the “Ontology Database of the Emotional Lexicon of the Dalian University of Technology” compiled by Xu et al. (2008), this study divides the emotional intensity of vocabulary into five levels ranging from low to high. Emotional polarity is divided into two categories (positive and negative). Positive emotions include joy, contentment, and shock, and negative emotions include anger, sorrow, fear, and disgust. In a tweet title, the word with the highest level of emotional intensity, along with its polarity, represents the emotional intensity and emotional polarity of the entire tweet title.

The signaling theory points out that in the entire process from the initial sending to the final understanding of the signal, the number, frequency, intensity, and emotional tendency of the signal will affect its visibility and effectiveness to the audience (Anderson, 1981; Bergh et al., 2014). The limited attention capacity model and research in the field of psychology have shown that individuals will assign different weights and attention resources to information or stimuli of different polarities (positive or negative). And compared with positive information, negative information is often given prioritized attention and will be processed first and thus has a stronger capacity to spread (Skowronski and Carlston, 1989; Zhu et al., 2014; Hornik et al., 2015). Research by Yang et al. (2007) found that fear-related information will break through noise suppression faster than other types of information to enter an individual’s consciousness. For different types of WeChat public accounts, the emotional intensity and polarity of the tweet titles also have different effects on the audience’s click-and-read behaviors. Specifically, for WeChat public accounts with the purpose of knowledge communication, general emotional information is often not the focus of the audience. In particular, tweet titles with positive emotional polarity will not necessarily attract more attention resources from the audience. Also, the level of the emotional intensity of these tweet titles will not have a significant impact on the audience’s click-and-read behaviors. Though a title with negative emotional polarity may become a stimulus, an emotional stimulus that is too strong will make the rational audience doubt the professionalism and authenticity of the tweet, which is not conducive to triggering the audience’s click-and-read behaviors. For WeChat public accounts with the purpose of information releasing, since the audience is mostly sensitive to emotional information, both positive and negative emotional information will become the emotion-stimulating signals for the audience. But for information with different emotional polarity, different levels of emotional intensity will have a different impact on the audience’s click-and-read behaviors. To be specific, negative tweet titles with high emotional intensity will cause the audience to generate more curiosity and devote more attention resources, thus triggering more click-and-read behaviors. Positive tweet titles with high emotional intensity generally do not strengthen the curiosity of the audience, and excessive emotional stimulation will make the audience doubt the authenticity of the tweet. Hence, the following assumptions are proposed:

*Hypothesis 3a: For WeChat public accounts serving the purpose of knowledge communication, negative tweet titles with a lower level of emotional intensity are more likely to trigger the click-and-read behaviors of the audience than negative tweet titles with a higher level of emotional intensity*.

*Hypothesis 3b: For WeChat public accounts serving the purpose of knowledge communication, the level of the emotional intensity of positive tweet title has no significant effect on the click-and-read behaviors of the audience*.

*Hypothesis 3c: For WeChat public accounts serving the purpose of information releasing, negative tweet titles with a higher level of emotional intensity are more likely to trigger the click-and-read behaviors of the audience than negative tweet titles with a lower level of emotional intensity*.

*Hypothesis 3d: For WeChat public accounts serving the purpose of information releasing, positive tweet titles with a lower level of emotional intensity are more likely to trigger the click-and-read behaviors of the audience than positive tweet titles with a higher level of emotional intensity*.

## Methodology

### Research Sample

To study the impact of emotional information of the tweet title on the click-and-read behaviors of the audience for different types of WeChat public accounts, it is necessary to control the influence of such factors as the number of followers, popularity, and reputation of the public account; also, a certain volume of tweet data is required. Therefore, this study selects one public account from each of the two categories of WeChat public accounts (knowledge communication and information releasing) with comparable numbers of followers, degrees of popularity, and volume of tweets, and obtains their real tweet data backstage. After obtaining the data, two graduate students were asked to screen the data separately, and deleting abnormal data, such as voting activities, holiday blessings, and other data in the data, respectively. Then, the third student makes judgment on the different parts of the processing results of the first two students, and finally, the researcher determines the final database. In consideration of the external influence during our sampling period, we believe that the main reason affecting the general mood of the audience is the occurrence of specific group events. Therefore, after consulting the data of 2019, 2020, we have found that there was no particularly prominent group event during these 2years. Thus, it can be inferred that the external influence is similar during these 2years. Considering the balance of data volume of the two public accounts, we have selected two specific periods for sampling the two public accounts with a similar volume of data. Specifically, we selected the public account of a new think tank in a university in Zhejiang Province, China, as the sample for the WeChat public account serving the purpose of knowledge communication. We selected the public account of a student union of a university in Zhejiang Province, China, as the sample for the WeChat public account serving the purpose of information releasing. This study used the real tweet data of these two public accounts from January 1, 2019, to December 30, 2020.

### Data Processing

This research utilizes programs written in the Python language to match characters in tweet titles in the sample WeChat public accounts with characters in the emotional lexicon, select the emotional vocabulary in the tweet titles, and use sentiment value algorithms to intelligently obtain values of variables, such as emotional presence, emotional complexity, emotional intensity, and emotional polarity. The variable of the click-and-read behavior of the audience is the actual number of times when the tweet was clicked and read. This study encodes the emotional information of tweet titles by capturing emotional vocabulary from “Ontology Database of the Emotional Lexicon of the Dalian University of Technology” compiled by Xu et al. (2008), in which emotional information in Chinese vocabulary or phrases were depicted and annotated from different angles such as emotional category and emotional intensity. The lexical ontology library that we have used is the lexical ontology library of emotions (version 1.0). In this thesaurus, emotional vocabulary is divided into seven categories with 21 subcategories, and the emotional polarity (positive and negative) of each subcategory is clearly marked (see Table 1). In this study, the criterion for determining the presence or absence of emotion in the tweet title is whether the title contains a word from the emotional vocabulary from the thesaurus, where 1 indicates the emotional presence and 0 indicates emotional absence. The criterion for determining the emotional complexity of a tweet title is the number of emotional vocabulary words included in the title, which are counted as 1, 2 … *n* from low to high. The criterion for determining the emotional intensity of a tweet title is the emotional intensity value of the emotional vocabulary with the highest emotional intensity in the title, counted with five levels from low to high as 1, 3, 5, 7, and 9, respectively. The criterion for determining the emotional polarity of the tweet title is the emotional polarity corresponding to the vocabulary with the highest emotional intensity in the title, where 1 indicates positive and −1 indicates negative.

**Table 1:** The classification of emotions.

No.	Category	Subcategory	Examples
1	Joy	Happy (PA)	Amusement, merit, smiling, rejoice
2		Peace of mind (PE)	Relieved, reassured, relax, conscientious
3	Contentment	Respect (PD)	Admire, venerate, solute, awe-inspiring
4		Praise (PH)	Handsome, excellent, reasonable, realistic
5		Believe (PG)	Trust, reliable, dependable, undoubted
6		Favor (PB)	Admire, cherish, love, at first sight, favorite
7		Wish (PK)	Longing, blessing, longevity
8	Anger	Rage (NA)	Indignant, annoy, furious, mad
9	Sorrow	Afflicted (NB)	Sad, grief, heartbroken, depressed
10		Disappointed (NJ)	Regret, despair, frustrate, discouraged
11		Guilt (NH)	Remorse, confession, unsatisfied, conscience
12		Yearn (PF)	Miss, lovesickness, indulgence, pine
13	Fear	Panic (NI)	Flurry, flustered, overwhelmed, confused
14		Dread (NC)	Timid, scared, terrified, horrified
15		Shame (NG)	Shy, disgraced, bashful, ashamed
16	Disgust	Bored (NE)	Depressed, irritable, upset, self-seeking trouble
17		Loathe (ND)	Disgust, antipathy, ignominious, hatred
18		Belittle (NN)	Stiff, vanity, disorderly, cruel
19		Jealous (NK)	Envy, sour, jealousy
20		Doubt (NL)	Suspect, distrust, discredit, disbelieve
21	Shock	Amazed (PC)	Strange, miracle, startled, dumbfounded

*Source: “Ontology Database of the Emotional Lexicon of Dalian University of Technology” (Xu et al., 2008)*.

## Empirical Analysis

### Descriptive Statistical Analysis

After removing the abnormal data, we selected a total of 1,465 real tweet data records within the relevant statistical period of this study for both types of the WeChat public accounts (knowledge communication and information releasing; see Table 2). Among the 694 tweets of the WeChat public accounts for knowledge communication, there are 220 titles without emotional vocabulary, accounting for 31.70% of the total, with an average of 399 occurrences of click-and-read. There are 68 titles with negative emotion, accounting for 9.80% of the total, with an average of 342 occurrences of click-and-read. There are 406 titles with positive emotion, accounting for 58.5% of the total, with an average of 361 occurrences of click-and-read. Among the 771 tweets of the WeChat public accounts for information releasing, there are 381 titles without emotional vocabulary, accounting for 49.42% of the total, with an average of 406 occurrences of click-and-read. There are 56 titles with negative emotion, accounting for 7.26% of the total, with an average of 551 occurrences of click-and-read. There are 334 titles with positive emotion, accounting for 43.32% of the total, with an average of 631 occurrences of click-and-read.

**Table 2:** Descriptive statistics of emotional information in tweet titles of WeChat public accounts for knowledge communication and information releasing.

Type	Emotional presence	Number of tweets	Percentages	Average times of click-and-read	The standard deviation of click-and-read times	Minimum value of click-and-read times	Maximum value of click-and-read times
Knowledge communication	Absent	220	31.70	399	285.938	8	1,314
	Negative	68	9.80	342	349.942	6	1,307
	Positive	406	58.50	361	312.490	4	2,347
Information releasing	Absent	381	49.42	406	446.986	3	2,648
	Negative	56	7.26	551	454.729	6	2,204
	Positive	334	43.32	631	576.866	10	3,151

### Analysis of the Influence of the Emotional Presence of Tweet Titles on the Click-and-Read Behaviors of the Audience for Different Types of WeChat Public Accounts

In this study, an independent sample *t* test was performed on the tweet data of the two types of WeChat public accounts to analyze the impact of emotional presence in tweet titles on the audience’s click-and-read behavior. The results are shown in Table 3. For WeChat public accounts of knowledge communication type, there is no significant difference in the impact of presence or absence of emotion in tweet titles on the audience’s click-and-read behavior (*T* value=1.601, *p*=0.110>0.05); therefore, H1a is effectively verified. For WeChat public accounts of information-releasing type, there are significant differences between the impact of emotional titles and the impact of non-emotional titles on the audience’s click-and-read behavior (*T* value=−5.827, *p*=0.000<0.05), and the mean of the occurrences of click-and-read for WeChat public accounts with emotional tweet tile (620) are greater than that for WeChat public accounts without emotional tweet tile (406); therefore, H1b is effectively verified.

**Table 3:** Independent sample t test results of the impact of the emotional presence of the tweet title on audience’s click-and-read behavior.

	Emotional presence		Emotional absence		*T* value	*p*
	Mean	Standard deviation	Mean	Standard deviation		
The type of knowledge communication – times of click-and-read	358	317.813	399	285.938	1.601	0.110
The type of information releasing – times of click-and-read	620	561.157	406	446.986	−5.827	0.000

### Analysis of the Influence of the Emotional Complexity of Tweet Titles on the Click-and-Read Behaviors of the Audience for Different Types of WeChat Public Accounts

This study uses regression analysis to analyze the effect of the emotional complexity of the tweet title on the audience’s click-and-read behavior of the two types of WeChat public accounts and constructs two corresponding regression models (see Table 4). The analysis results of regression model 1 show that for the WeChat public accounts for knowledge communication, the emotional complexity of the tweet title has no significant impact on the audience’s click-and-read behavior (*β*=−0.026, *p*=0.571>0.05); therefore, H2a is effectively verified. The analysis results of regression model 2 show that for the WeChat public accounts for information releasing, the emotional complexity of the tweet title has a significant negative impact on the audience’s click-and-read behavior (*β*=−0.141, *p*=0.005<0.05); therefore, H2b is effectively verified.

**Table 4:** Regression analysis results of the impact of the emotional complexity of the tweet title on audience’s click-and-read behavior.

Model	Independent variable	Dependent variable	Standardized coefficient *β*	*T* value	*p*	*F* value	Coefficient of determination *R*^2^
1	The type of knowledge communication – emotional complexity	Times of click-and-read	−0.026	−0.567	0.571	0.321	0.001
2	The type of information releasing – emotional complexity	Times of click-and-read	−0.141	−2.801	0.005	7.843	0.020

### Analysis of the Interactive Influence of Emotional Intensity and Emotional Polarity of Tweet Titles on the Click-and-Read Behaviors of the Audience in Different Types of WeChat Public Accounts

This study uses regression analysis to analyze the interactive effect of emotional intensity and emotional polarity of the tweet title on the audience’s click-and-read behavior of the two types of WeChat public accounts and constructs four corresponding regression models (see Table 5). The analysis results of regression model 1 show that for the WeChat public account of the knowledge communication type, the emotional intensity of the negative tweet title has a significant negative impact on the audience’s click-and-read behavior (*β*=−0.355, *p*=0.003<0.05); therefore, H3a is effectively verified. The analysis results of regression model 2 show that in the WeChat public account of the knowledge communication type, the emotional intensity of the positive tweet title has no significant effect on the audience’s click-and-read behavior (*β*=0.035, *p*=0.479>0.05); therefore, H3b is effectively verified. The analysis results of regression model 3 show that for the WeChat public account of the information-releasing type, the emotional intensity of the negative tweet title has no significant effect on the audience’s click-and-read behavior (*β*=0.192, *p*=0.157>0.05); therefore, H3c is not verified. The analysis results of regression model 4 show that for the WeChat public account of the information-releasing type, the emotional intensity of the positive tweet title has a significant negative impact on the audience’s click-and-read behavior (*β*=−0.236, *p*=0.000<0.05), therefore, H3d is effectively verified.

**Table 5:** Regression analysis results of the impact of emotional intensity and emotional polarity of the tweet title on audience’s click-and-read behavior.

Model	Independent variable	Dependent variable	Standardized coefficient *β*	*T* value	*p*	*F* value	Coefficient of determination *R*^2^
1	The type of knowledge communication – negative emotional intensity	Times of click-and-read	−0.355	−3.082	0.003	9.501	0.126
2	The type of knowledge communication – positive emotional intensity	Times of click-and-read	0.035	0.709	0.479	0.503	0.001
3	The type of information releasing – negative emotional intensity	Times of click-and-read	0.192	1.434	0.157	2.058	0.037
4	The type of information releasing – positive emotional intensity	Times of click-and-read	−0.236	−4.418	0.000	19.516	0.056

## Conclusion and Discussion

### Conclusion

First, the emotional presence of the tweet title has a varying impact on the audience’s click-and-read behavior for different types of WeChat public accounts. Our results show that for the WeChat public account for knowledge communication, the emotional presence of the tweet title has no significant effect on the audience’s click-and-read behavior. On the contrary, for the WeChat public account for information releasing, tweet titles with the presence of emotion are more likely to trigger the audience’s click-and-read behavior. Thus, based on the limited attention capacity model and the signaling theory, in the face of massive WeChat public account tweets, audiences with limited attention resources may regard the emotional vocabulary in the tweet titles as a stimulating signal to decide whether to click-and-read. Due to the differences in reading motivation and information focus of the audiences who subscribe to different types of WeChat public accounts, the emotional vocabulary in the tweet title is not effective for all audiences. To some extent, it complements and verifies the research results of Schoefer and Ennew (2005), Phelps et al. (2006), Li and Liu (2018).

Second, for different types of WeChat public accounts, the emotional complexity of the tweet title has a varying impact on the audience’s click-and-read behavior. Our results show that for the WeChat public account for knowledge communication, the emotional complexity of the tweet title has no significant effect on the audience’s click-and-read behavior. On the contrary, for the WeChat public account for information releasing, tweet titles with lower emotional complexity are more likely to trigger the audience’s click-and-read behavior. This is similar to the conclusion of Miller (1976). This shows that although the emotional vocabulary in the tweet titles of the WeChat public accounts may stimulate the click-and-read behaviors of some audiences, especially those who prefer novelty and entertainment, it is not true that the richer the emotional vocabulary in the tweet titles the better. Tweet titles with complex emotional information will, to a certain extent, weaken the audience’s perception of novelty and prevent them from making further click-and-read decisions.

Third, Emotional intensity and emotional polarity of the tweet title have different interactive effects on the audience’s click-and-read behavior for different types of WeChat public accounts. To some extent, this study complements the studies of some scholars, such as Zhu et al. (2014), Anderson (1981), Skowronski and Carlston (1989), and Bergh et al. (2014). Our results show that for the WeChat public accounts for knowledge communication, the emotional intensity of negative tweet titles has a significant negative impact on the audience’s click-and-read behavior, and the emotional intensity of positive tweet titles has no significant impact on the audience’s click-and-read behavior. For the WeChat public account for information releasing, the emotional intensity of negative tweet titles has no significant effect on the audience’s click-and-read behavior, and the emotional intensity of positive tweet titles has a significant negative impact on the audience’s click-and-read behavior. It can be seen that regardless of the type of WeChat public account or the emotional polarity of the tweet titles, the excessive emotional intensity will cause the audience to doubt the authenticity of the tweets. Therefore, it is not necessarily that the higher the emotional intensity the better. At the same time, the reason why H3c has not been effectively verified may be that for the audience who prefers novelty and entertainment, tweet titles with negative emotional polarity may act as a strong emotional stimulating signal. Therefore, the focus of the audience is mainly on the emotional polarity of the tweet title rather than the emotional intensity, which may have caused the result that the impact of emotional intensity on the audience’s click-and-read behavior is no longer significant.

### Theoretical Contribution and Implications

This study has the following theoretical contribution. First, the selection of tweet titles of the WeChat public account as the research object has enriched and expanded the research field of social media marketing. WeChat public accounts are currently the most popular carrier of social media marketing in China. There has been related research on the sharing, contents, text analysis, and marketing effects of information posted in the WeChat public accounts (Anjaria and Guddeti, 2014; Tago and Jin, 2018). However, less attention has been paid to the impact of tweet titles in public accounts on the audience’s click-and-read behaviors. Second, this paper further analyzes the impact of emotional factors such as emotional presence, emotional complexity, emotional intensity, and emotional polarity of tweet titles on the click-and-read behavior of the audience based on the existing studies of Zhao et al. (2012), Feng and Tang (2017), and Li and Liu (2018). The perspective of emotional expression taken in our analysis on the tweet title information of the WeChat public accounts has deepened and refined related research on the audience’s handling of social media information. Third, by applying the limited attention capacity model and the signaling theory, this study has constructed a basic theoretical analysis framework for further exploring the audience’s emotional information processing mechanism of tweet titles in WeChat public accounts. Based on the existing research of Bergh et al. (2014), Schmidt et al. (2014), and Xu et al. (2008), this paper takes multiple theoretical perspectives and studies the influence mechanism of the emotional information of tweet titles on the audience’s click-and-read behavior in different types of WeChat public accounts. We also conducted an empirical study using the real data of two types of WeChat public accounts which has, to some extent, provided theoretical basis and insights for follow-up research.

This study has also provided the following implications. We have provided some practical guidance and managerial inspiration for enterprises and other organizations to scientifically use the emotional expression of the tweet titles in WeChat public accounts to improve the effectiveness of information dissemination and marketing promotion.

First of all, great importance should be attached to the emotional expression of tweet titles in the WeChat public accounts. In particular, according to the results of this study that WeChat public accounts that mainly target audiences with a preference for novelty and entertainment (such as those serving the purpose of information releasing) should be encouraged to use emotionally expressive tweet titles to release emotionally stimulating signals to the target audience to increase the number of clicks on tweets, thus enhancing the effect of information dissemination and promotion.

Second, overly complex emotional expressions should be avoided as much as possible in tweet titles in WeChat public accounts. This study points out that when there is too much emotional vocabulary that appears in the title of a tweet, it may lead to the audience’s difficulty in making decisions and the tendency for them to verify the authenticity of the tweets. Instead, a single category of emotional vocabulary (such as joy, contentment, and anger) should be used to send a clear-cut stimulating signal to the target audience, to avoid the hindering of click-and-read behaviors of the audience.

Third, an appropriate level of emotional intensity should be stressed in tweet titles in the WeChat public accounts, and emotional intensity should be matched with the type and emotional polarity of the public account. In general, it is not necessarily true that the level of the emotional intensity of tweet titles in various types of WeChat public accounts should be as high as possible. Therefore, over-usage of high-intensity emotional vocabulary in tweet titles should be avoided to eliminate or lower the chances that the audience may question the authenticity of the tweet and give up clicking to read. The intensity of emotional vocabulary in the titles should also be matched with the type and emotional polarity (positive or negative) of the public account to maximize the promoting effect of the emotional expression of tweet titles on the audience’s click-and-read behavior.

### Limitations and Future Prospects

This study has the following limitations. First, we only analyzed the impact of various emotional information of the tweet title in different types of WeChat public accounts on the click-and-read behaviors of the audience and did not take into consideration the influence of other factors such as the number of words in the title, the content of the title, the picture shown with the title, and the timing when the tweet is posted. Second, the fact that only 1,465 real tweet data of two types of WeChat public accounts were studied and that we have limited the types of WeChat public accounts concerned, in addition to that we only had a relatively smaller sample size of tweet titles with negative emotion, may affect the applicability and generality of the research conclusions. The two selected accounts might not be sufficiently representative since their audients are both college students. Third, when studying the interactive effect of emotional intensity and emotional polarity of the tweet title on the audience’s click-and-read behavior, the influence of different levels of emotional intensity on the audience’s click-and-read behavior was analyzed only under the same emotional polarity. Forth, the research results of the impact of emotional expression in titles on behaviors have very rich application scenarios, such as reading apps, virtual community and so on. And the impact of emotional polarity of titles on behaviors has attracted the attention of some businesses who has started to explore its potential application in practice. For example, Netease has launched a program where it charges merchants by providing them with predicting services based on the impact of emotional polarity of titles and headlines of the merchants on the behavior of their consumers. However, most of these services are very premature since they only start from the perspective of a single emotional polarity and rarely consider such factors as emotional intensity and emotional complexity, which has provided clues for future research to explore different app or software scenarios. In addition, future research can also explore more possibilities in cultural contexts other than the Chinese one to verify the universality and exploit deeper implication of the research results. Fifth, it would be better if more advanced sentiment analysis techniques are applied. In future research, the above issues should be more comprehensively considered, so a more in-depth and systematic analysis can be carried out on the click-and-read behavior concerning WeChat public account tweets from an emotional perspective. And we will also consider other research contexts by exploring other different apps/soft wares as well.

## Data Availability Statement

The original contributions presented in the study are included in the article/supplementary material, further inquiries can be directed to the corresponding author.

## Author Contributions

TC and LL analyzed the data and wrote the manuscript. JF and TC conceived the idea of the manuscript, designed the research, and revised the manuscript. LL provided constructive suggestions to improve the research. All authors have read and approved the final manuscript.

## Funding

This research is part of the key program financially supported by the National Social Science Foundation of China (Grant number: 20AGL019).

## Conflict of Interest

The authors declare that the research was conducted in the absence of any commercial or financial relationships that could be construed as a potential conflict of interest.

## Publisher’s Note

All claims expressed in this article are solely those of the authors and do not necessarily represent those of their affiliated organizations, or those of the publisher, the editors and the reviewers. Any product that may be evaluated in this article, or claim that may be made by its manufacturer, is not guaranteed or endorsed by the publisher.
